# Adverse Events of Monoclonal Antibodies Used for Cancer Therapy

**DOI:** 10.1155/2015/428169

**Published:** 2015-05-05

**Authors:** Mei Guan, Yan-Ping Zhou, Jin-Lu Sun, Shu-Chang Chen

**Affiliations:** ^1^Department of Oncology, Peking Union Medical College Hospital, Peking Union Medical College and Chinese Academy of Medical Sciences, Beijing 100730, China; ^2^Department of Allergy, Peking Union Medical College Hospital, Peking Union Medical College and Chinese Academy of Medical Sciences, Beijing 100730, China

## Abstract

In 1997, the first monoclonal antibody (MoAb), the chimeric anti-CD20 molecule rituximab, was approved by the US Food and Drug administration for use in cancer patients. Since then, the panel of MoAbs that are approved by international regulatory agencies for the treatment of hematopoietic and solid malignancies has continued to expand, currently encompassing a stunning amount of 20 distinct molecules for 11 targets. We provide a brief scientific background on the use of MoAbs in cancer therapy, review all types of monoclonal antibodies-related adverse events (e.g., allergy, immune-related adverse events, cardiovascular adverse events, and pulmonary adverse events), and discuss the mechanism and treatment of adverse events.

## 1. Introduction

Engineered monoclonal antibodies (MoAbs) represent a significant addition to the therapeutic armamentarium for a variety of malignancies. Adverse events (AEs) of these new regimens are described to be mild compared with those of classical chemotherapy. Twenty MoAbs are currently registered and approved for the treatment of a range of different cancers. These MoAbs are specific for 11 targets. Five of these molecules are directed against the B-lymphocyte antigen CD20, 3 against human epidermal growth factor receptor 2 (HER2 or ErbB2), 3 against the epidermal growth factor receptor (EGFR), 2 against vascular endothelial growth factor (VEGF), and 1 each against epithelial cell adhesion molecule (EpCAM), CD30, CD52, tumor necrosis factor (ligand) superfamily member 11 (TNFSF11, also known as RANKL), cytotoxic T lymphocyte-associated protein 4 (CTLA-4), programmed death 1 protein (PD-1) and interleukin-6 (IL-6) are summarized in Table 1. Common adverse events (AEs) include allergy (rash, infusion reactions), diarrhea, hypertension, proteinuria, hypothyroidism, and hepatotoxicity. Certain toxicities are caused by on-target, mechanism-associated effects, which can be stratified by whether or not the targets are relevant to response. Other toxicities are off-target and may be caused by immune reactions or toxic metabolites. Here, we review monoclonal antibodies-related AEs and management of patients displaying these reactions.

## 2. Drug Allergy

**Figure 1:**
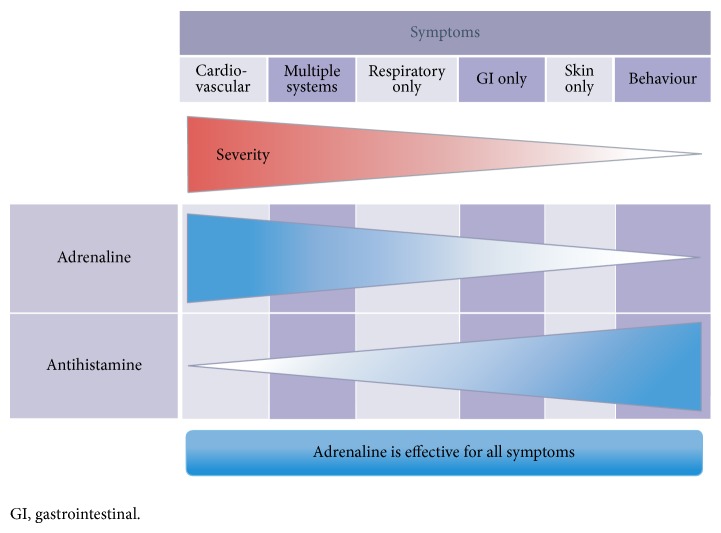
Symptoms associated with anaphylaxis.

Historically, immunologic reactions have been divided into four categories (I to IV) according to the Gell and Coombs system. Drugs are most commonly implicated in type I reactions. These reactions, mediated by IgE antibodies are also known as anaphylactic hypersensitivities and are relatively uncommon after administration of MoAbs. Immediate hypersensitivity may affect a single organ such as the nasopharynx (allergic rhinitis), eyes (conjunctivitis), mucosa of mouth/throat/tongue (angioedema), bronchopulmonary tissue (asthma), gastrointestinal tract (gastroenteritis), and skin (urticaria, eczema) or multiple organs (anaphylaxis). They cause symptoms that range from minor itching and inflammation to death. Symptoms associated with anaphylaxis are shown in Figure 1 [1]. Anaphylaxis has been reported for cetuximab, rituximab, trastuzumab, pertuzumab, obinutuzumab, ofatumumab, tositumomab, and ibritumomab, and these last two MoAbs have also been reported to cause bronchospasm and angioedema [2–6].

A high prevalence of hypersensitivity reactions to cetuximab have been reported in some areas of the United States. In most subjects who had a hypersensitivity reaction to cetuximab, IgE antibodies against cetuximab were present in serum before therapy [7–10]. The antibodies are specific for an oligosaccharide, galactose-*α*-1,3-galactose, which is present on the Fab portion of the cetuximab heavy chain. The presence of such antibodies is predictive of anaphylaxis, and pretreatment testing would help in minimizing the risk of anaphylaxis associated with cetuximab [11].

### 2.1. Standard Infusion Reactions (SIR)

Nearly all MoAbs share a risk for standard infusion reactions (SIR), but certain drugs (e.g., rituximab, cetuximab, alemtuzumab, ramucirumab, obinutuzumab, and ofatumumab) are associated with a high enough risk to warrant special precautions. The most common symptoms and signs are dyspnea, nausea, headache, and abdominal pain. Most reactions are mild; only approximately 0.3% of patients have serious infusion reactions with features of anaphylaxis (bronchospasm, hypotension, and angioedema). Standard infusion reactions typically develop within 30 minutes to two hours after the initiation of drug infusion, although symptoms may be delayed for up to 24 hours. The majority of reactions occur after the first or second exposure to the agent, but between 10 and 30% occur during subsequent treatments. Rituximab, obinutuzumab, and trastuzumab induce the highest incidence of SIR. In general, the incidence of MoAb induced IR varies from 15–20% for cetuximab (3% grade 3/4) and 40% for trastuzumab first infusion (<1% grade 3/4) to 77% for rituximab first infusion (10% grade 3/4). Infusion reactions are markedly less common after the initial infusion [12]. The manufacturer reports a frequency of 77, 30, and 14% during the first, fourth, and eighth infusions of rituximab, respectively. The incidence of IR to the humanized MoAbs bevacizumab, panitumumab and nimotuzumabis is significantly lower at <3% (0.2% grade 3/4). The mechanism underlying MoAb-related infusion reactions remain unclear, but most are thought to be related to antigen-antibody interactions precipitating cytokine release [5, 6]. It is the most predictable reaction that occurs with rituximab, and is thought to be caused by the interaction of the drug with the target antigen (CD20) on circulating cells, followed by cytokine release from lymphocytes. Evidence for this mechanism includes the observation that severe and fatal reactions have typically occurred in patients with high numbers of circulating lymphocytes bearing the target antigen [5].

### 2.2. Serum Sickness (A Delayed Type III Allergic Reaction)

Serum sickness has been reported with rituximab. Symptoms include fever and arthralgia with a morbilliform skin eruption that often has acral accentuation. The reaction typically develops one to two weeks after treatment and is accompanied by laboratory evidence of complement activation (depressed C3 and C4 levels) and tissue inflammation (elevated erythrocyte sedimentation rate and C-reactive protein) [13]. Chimeric MoAbs have the potential to induce serum sickness. Recently, it has been reported that rituximab-induced serum sickness-like reactions occur in 1–20% of patients [14].

### 2.3. Treatment

#### 2.3.1. Prevention

Pharmacologic prophylaxis with a histamine H1 receptor antagonist is recommended prior to each infusion of ramucirumab. Pharmacologic prophylaxis with antihistamines and acetaminophen with or without a glucocorticoid is suggested for high-risk agents (i.e., rituximab, first infusion of cetuximab in a patient who resides in a high-risk area and intravenous alemtuzumab). Despite premedication, clinicians must be prepared for an infusion reaction to occur during each drug administration [6, 15].

#### 2.3.2. Mild to Moderate SIR

If the reaction is limited to mild or moderate symptoms of SIR (grades 1 or 2), without features suggestive of anaphylaxis, drug infusion should be temporarily stopped and assessment of airway, breathing, circulation, and mentation should rapidly occur. IV administration of 50 mg of diphenhydramine and 650 mg of acetaminophen may provide symptomatic relief. Once symptoms have resolved, resumption of the drug infusion at a slower rate may permit treatment continuation with close monitoring [6].

#### 2.3.3. Severe SIR or Anaphylaxis

Severe SIR (grades 3/4) or reactions of any severity with any features of anaphylaxis (e.g., generalized urticaria, wheezing, hypotension, and angioedema) require prompt recognition and treatment. Clinical criteria for diagnosing anaphylaxis can be seen in Table 2. Recommendations for emergency management are shown in Table 3 [1]. The first line drug treatment is adrenaline.

#### 2.3.4. Rechallenge

Once the acute event has subsided, the issue of rechallenge must be addressed. Patients with severe infusion reaction or anaphylaxis to cetuximab can be safely switched to pannitumumab. The decision to attempt retreatment depends upon the drug, the severity of the reaction, the cancer being treated, and whether there are reasonable treatment alternatives [5, 6].

#### 2.3.5. Desensitization

Experience with desensitization to MoAbs is relatively limited. At some institutions, these are only performed by allergy specialists [63].

## 3. Immune-Related Adverse Events (irAEs)

Ipilimumab is a monoclonal antibody that targets CTLA-4, thus unleashing an immune reaction against the tumor. CTLA-4 is a surface protein expressed on activated and regulatory T cells and is upregulated in malignancy [56]. Immune-related adverse events (irAEs) can occur at any point during treatment with ipilimumab, but often first present around the third or fourth dose. The incidence of hypophysitis due to ipilimumab has been reported to range from 0 to 17% in clinical trials, though the mechanism of pituitary injury remains unknown. Other immune-related adverse events include hepatotoxicity, or failure of the thyroid gland (autoimmune thyroiditis), the adrenal gland, and gonadal axis, and enterocolitis, which can be serious or life-threatening (any grade 30%–35%, grade 3–5 diarrhea 5%–8%) [57]. It remains unclear whether the effects result from T cells specifically acting against antigens shared by tumor and normal cells or from the concomitant activation of multiple T cell populations with separate antihost and antitumor activity [64, 65]. Current recommendations include baseline TSH and free T4 and monitoring every 3 weeks during ipilimumab treatment and every 2-3 months following completion. For patients with severe or life-threatening grade 3/4 AEs, treatment with ipilimumab should be permanently discontinued and high doses of corticosteroids (prednisone 1 to 2 mg/kg/day or equivalent) are indicated [56].

Radioimmunotherapy (RIT) refers to the use of monoclonal antibodies that are linked to radioisotopes (e.g., yttrium-90). Two drugs have been approved by the United States FDA to be used in treatment of relapsed or refractory CD20 positive, low-grade, follicular, or transformed non-Hodgkin's lymphoma (NHL). Ibritumomabtiuxetan (Zevalin) is a murine anti-CD20 monoclonal antibody conjugated to yttrium-90 [2]. Tositumomab (Bexxar) is a murine anti-CD20 monoclonal antibody conjugated with radioactive iodine-131 [3]. RIT may also lead to hypothyroidism; patients should receive thyroid-blocking medications beginning at least 24 hours prior to tositumomab and continued for 2 weeks after the therapeutic dose. Nivolumab is a humanized monoclonal antibody that targets the PD-1 protein. Immune-related adverse events are the most common side effects, and the skin and gastrointestinal tract are the most often affected organ systems and less frequently hepatic, endocrine, and neurologic events occur [66, 67].

## 4. Cardiovascular AEs

Cardiac adverse events have occurred with specific MoAbs, including bevacizumab, trastuzumab, trastuzumabemtansine, pertuzumab, ofatumumab, and rituximab [5, 22, 38, 45, 68, 69].

### 4.1. Hypertension

VEGF plays a key role in the maintenance of vascular homeostasis via mediation of the production of the vasodilator nitric oxide and decrease of vascular resistance through the generation of new blood vessels [68, 70–72]. The overall incidence of bevacizumab-induced hypertension is approximately 12 to 34%, with severe hypertension in 5 to 18%. Hypertension has been proposed to be a clinical biomarker of antitumor activity [73]. The incidence rate of hypertension for ramucirumab was lower than bevacizumab (all grades: 6%; grades 3/4: 8%) [74].

### 4.2. Arterial and Venous Thromboembolism

An increased risk for arterial thromboembolic events (ATEs) has been linked to the use of bevacizumab and ramucirumab [23, 24, 28, 75]. The overall incidence of bevacizumab-induced thromboembolism is ≤21% (grades 3/4: 15%) and consists of venous thromboembolism (all-grade: 8%; grades 3/4: 5% to 7%) and arterial thrombosis (all-grade: 6%; grades 3/4: 3%). In a pooled analysis of 1,745 patients, of whom 963 were treated with bevacizumab (24% breast cancer), the incidence of thromboembolic events was 4% in patients treated with bevacizumab plus chemotherapy, and 2% in those treated with chemotherapy alone [75]. Ramucirumab-induced arterial thrombosis (including myocardial infarction, cardiac arrest, cerebrovascular accident, and cerebral ischemia) was seen in 2% patients [28].

### 4.3. Congestive Heart Failure (CHF)

A meta-analysis of five randomized trials involving a total of 3,784 metastatic breast cancer patients analyzed the incidence of congestive heart failure (CHF) when using chemotherapy with or without bevacizumab. The incidence of high-grade CHF was 1.6% in patients treated with bevacizumab and 0.4% in patients who did not receive this drug [24]. In NSABP B31, asymptomatic decrease in LVEF occurred in 14% of patients, requiring discontinuation of trastuzumab [22]. Endomyocardial biopsy was performed in a limited number of patients exposed to trastuzumab and demonstrated no significant abnormalities [31]. The incidence of severe CHF in the trastuzumab adjuvant studies is in the range of 1% to 4%. In the Herceptin Adjuvant trial (HERA), with 3.6 years of median followup, all cases of severe CHF occurred during trastuzumab treatment; however, the cardiac performance of the majority of affected patients improved when trastuzumab was withdrawn [32]. The incidence of CHF in older patients treated with trastuzumab is expected to be higher than in the overall population evaluated in large clinical trials [33]. Combining anti-HER2 and anti-VEGF drugs has consequently emerged as an important strategy to optimize the targeted treatment of breast cancer. The bevacizumab plus trastuzumab combination was evaluated in 50 heavily pretreated metastatic breast cancer patients [76]. This combination was associated with a 30% incidence of asymptomatic LVEF decrease, 2% grade 4 LVEF decrease, and 36% incidence of hypertension. In phases I–III of trials of pertuzumab, cardiac dysfunction was seen in 4.5–14.5% of patients with pertuzumab treatment and cardiac dysfunction was usually grade 1/2 [39]. Cardiotoxicity of pertuzumab was usually reported in combination with trastuzumab and no additive cardiotoxicity was reported with addition of pertuzumab to trastuzumab [38]. A phase II study evaluated trastuzumab-DM1 in 107 patients pretreated with anthracyclines, trastuzumab, taxanes, capecitabine, and lapatinib. Reduction in LVEF was observed in two patients [69].

### 4.4. Hemorrhage

All VEGF-targeted agents have been associated with an increased risk of hemorrhage. This is most commonly grade 1 epistaxis, though more serious, and in some cases, fatal hemorrhagic events have occurred, including hemoptysis (particularly in patients with squamous cell lung cancer), gastrointestinal bleeding, hematemesis, intracerebral hemorrhage, epistaxis, and vaginal bleeding. The overall risk of major bleeding is approximately 2 to 3%. A total of 12,917 patients from 17 RCTs treated with bevacizumab had a significantly increased risk of cerebrovascular events compared with patients treated with control medication, with a relative risk of 3.28 (95% CI, 1.97–5.48). The risks of CNS ischemic events and CNS hemorrhage were increased compared with control, with relative risk (RRs) of 3.22 (95% CI, 1.71–6.07) and 3.09 (95% CI, 1.36–6.99), respectively. Risk varied with the bevacizumab dose, with RRs of 3.97 (95% CI, 2.15–7.36) and 1.96 (95% CI, 0.76–5.06) at 5 and 2.5 mg/kg/week, respectively [25].

### 4.5. Treatment

To prevent cardiovascular adverse events, the physician should perform a pretreatment evaluation and screening, including formal risk assessment for potential cardiovascular complications. Preexisting hypertension should be identified and treated before using these agents. Caution and close serial monitoring of LVEF are warranted during therapy with bevacizumab in older adults and those with a history of hypertension, heart disease or anthracycline exposure. Cardiac troponin and amino-terminal fragment B-type natriuretic peptide (NT proBNP) have been the most frequently assessed biomarkers for cardiac injury and will be briefly described. Cardiac troponin is a medium-sized protein that regulates the cardiac contractile elements actin and myosin. The NT proBNP is useful for diagnosing cardiac failure in breathless patients but its utility for identifying subclinical cardiac pathology is unclear [77]. MoAbs should be discontinued for any severe ATE/VTE. Antiangiogenic therapy is associated with impairment of wound healing. It is recommended to withhold ramucirumab treatment prior to surgery. After surgery, clinical judgment dictates when to resume based on adequate wound healing. It is recommended that bevacizumab should be discontinued at least 28 days prior to surgery and should not be reinitiated for at least 28 days after surgery and until wound is fully healed [26, 28].

## 5. Pulmonary AEs

There are several complications that affect the lungs associated with the use of MoAbs, including interstitial lung disease (ILD), hemorrhage, trachea-esophageal fistula, and thromboembolic disease. Since the mechanisms underlying such lung injuries have generally not been uncovered, any classification on the basis of pathogenesis is difficult. Adverse events can be grouped into 4 main categories: interstitial pneumonitis and fibrosis, acute respiratory distress syndrome (ARDS), bronchiolitis obliterans organizing pneumonia (BOOP), and hypersensitivity reactions. Signs, symptoms, and clinical findings include dyspnea, cough, fatigue, and pulmonary opacities. Because signs and symptoms are generally nonspecific, the diagnosis usually remains one of exclusion [16, 34, 35, 43, 78, 79].

Once again, rituximab is the most implicated MoAb, inducing a heterogeneous spectrum of lung disorders. In 2003, the reported rate of possible drug-induced lung injury was <0.03% from >540,000 exposed patients. BOOP is the most common clinical diagnosis of rituximab-induced lung disease, followed by interstitial pneumonitis, ARDS, and hypersensitivity pneumonitis. Acute or subacute rituximab-induced lung disease, most notably organizing pneumonia, most likely reflects a hypersensitivity reaction to the potentially immunogenic chimeric anti-CD20 antibody. Arguments that support a hypersensitivity reaction include the recurrence and increasing severity of the symptoms from one infusion to the next, occurrence during the third month on average, responsiveness to steroid therapy (delayed onset 15 days after methylprednisolone infusion and favorable outcome with steroid therapy), rash and eosinophilia, BALF lymphocytosis, and histological pattern of organizing pneumonia in many patients [43]. Interstitial lung disease (ILD) has been reported in treatment with cetuximab and transtuzumab [16, 34].

Discontinuation of MoAb is advised in any patient who develops ILD or acute respiratory distress syndrome (ARDS) during treatment. Improvement following treatment with glucocorticoids has been reported; however, the role of glucocorticoid therapy in MoAb-induced pulmonary AEs has not been formally studied [35, 78, 79].

## 6. Proteinuria/Nephrotic Syndrome

Bevacizumab is associated with proteinuria, though rarely in the nephrotic range (>3.5 g/24 hours) and even more rarely associated with the nephritic syndrome [27, 80, 81]. Hypertension frequently accompanies proteinuria. Proteinuria is usually an asymptomatic event detected only through laboratory analysis. Reports of renal biopsies among patients with proteinuria receiving VEGF-targeted agents are sparse; when reported, the most common causative agent was bevacizumab. Histologic findings include thrombotic microangiography, collapsing glomerulopathy, and isolated reports of cryoglobulinemic and immune complex glomerulonephritis. The overall incidence of mild proteinuria in patients treated with bevacizumab ranges from 21 to up to 63%, but grade 3 or 4 proteinuria (defined as 3+ on dipstick, >3.5 g of protein/24 hours, or the nephrotic syndrome) affects approximately 2% of treated patients. The incidence is not higher in patients who receive shorter bevacizumab infusions (i.e., 10 versus 90 minutes) [82, 83]. The AEs of ramucirumab were lower than bevacizumab, with only  5.1% of patients experiencing proteinuria [28, 74].

Other less common renal problems that have been reported with bevacizumab include acute renal dysfunction and proliferative glomerulonephritis [27, 83].

Temporary cessation of bevacizumab is advised if protein excretion exceeds 2 g in 24 hours, and permanent discontinuation is appropriate for patients who develop the nephrotic syndrome [26].

## 7. Enterotoxicity

Enterocolitis, colitis, and gastrointestinal perforation (GIP) are common gastrointestinal AEs of MoAbs. In a study of pertuzumab monotherapy in patients with metastatic breast cancer, diarrhea of any grade developed in 48%, but it was severe (grade 3 or 4) in only 3% [39, 40]. A phase III comparison of best supportive care (BSC) with or without panitumumab reported diarrhea of any grade in 21% of patients receiving this MoAb (grade 3: 1%) compared to 11% with BSC alone (none grade 3) [18]. Cetuximab-related diarrhea is generally not severe, and while the rate of diarrhea of any grade was 12.7%, rates of grade 3 or 4 diarrhea in studies of single agent cetuximab have ranged from only 1.5 to 2% [17, 84].

All VEGF targeted therapies, including bevacizumab, can cause gastrointestinal perforation (GIP). GIP has been reported in patients treated with bevacizumab for a variety of malignancies, and has occurred in 0.3% to 2.4% of clinical study patients receiving bevacizumab. It can occur anywhere along the GI tract. Nongastrointestinal fistula formation also has been observed, most commonly within the first 6 months of treatment. Most cases occur within 50 days of treatment initiation. In order to minimize the risk of GIP and fistula formation, at least 28 days (preferably six to eight weeks) should elapse between surgery and last dose of bevacizumab, except in emergency situations [85].

## 8. Dermatologic/Cutaneous AEs

### 8.1. Papulopustular Acneiform Eruption

The most common cutaneous reaction pattern with the EGFR inhibitors is a diffuse papulopustular acneiform eruption, which is due to a role of EGFR in maintaining integrity of the skin. It is noted in more than two-thirds of patients receiving any of these agents (cetuximab, panitumumab) [17, 18]. The acneiform eruption is often dose-dependent, and typically begins early, within one week of treatment initiation. The lesions typically occur on the face, trunk, and extremities, sparing the palms and soles. Scaling of the interfollicular skin may also be present. Significant pruritus accompanies the cutaneous eruption in up to one-third of patients. Severity of the acneiform rash (all studies: 76% to 88%; grades 3/4: 1% to 17%; onset: ≤14 days) correlates with treatment response and prolonged survival in colorectal cancer patients treated with cetuximab [86]. In the ASPECCT trial, Grade 3-4 skin AEs occurred in 62 patients (13%) given panitumumab and 48 patients (10%) given cetuximab [19]. The skin AEs of nimotuzumab were very low, with only mild moderate skin rash observed [20].

### 8.2. Paronychial Inflammation

Paronychia involving the great toe is often the first sign, and secondary bacterial infection (often with* Staphylococcus aureus*) is not uncommon in patients treated with cetuximab [17, 87]. Other less common specific cutaneous reactions include the following: erythematous exanthem caused by cytomegalovirus, Stevens-Johnson syndrome, toxic epidermal necrolysis, and full thickness necrosis, which has been reported in a small number of patients treated with ipilimumab for metastatic melanoma. Treatment options include topical antibiotics, topical corticosteroids, and/or electrodessication for larger lesions. Temporary withholding of the drug is appropriate when the cutaneous complication is serious [57].

### 8.3. Treatment

Preventive/prophylactic management is recommended: hydrocortisone 1% combined with moisturizer, sunscreen, and doxycycline 100 mg bid for the first 6 weeks. Sunlight may exacerbate skin reactions (limit sun exposure). Treatment include the following: alclometasone 0.05% cream or fluocinonide 0.05% cream or clindamycin 1%, and doxycycline 100 mg bid or minocycline 100 mg daily or isotretinoin at low doses (20–30 mg/day) [88].

### 8.4. Mucositis/Stomatitis

Mucositis or stomatitis is a frequent oral complication for cetuximab (grades 3/4: 0.9%). It mostly affects the nonkeratinized labial and buccal mucosa, the mucosa of the tongue, of the floor of the mouth, and the soft palate and appears 9–16 days after treatment initiation, as this is the epithelial cell turnover time [17, 86]. Stomatitis has been reported with bevacizumab (grades 1/2: 23%) [89]. Tositumomab has a higher rate of severe mucositis than rituximab (52 versus 18%) [50]. Other dermatologic toxicities include the following: maculopapular, erythematous rash, skinxerosis, pruritus, and Stevens-Johnson syndrome. Changes of the nails include pitting, discoloration, and onycholysis, with partial or complete loss of nails [17, 86, 87].

## 9. Cytopenia

The most profound side effect of radioimmunotherapy (RIT) is potentially prolonged and significant cytopenia with cell count nadirs ranging from four to nine weeks posttherapy with recovery one to four weeks postnadir. The most common cytopenias are leukopenia and thrombocytopenia, which are easily managed in the majority of patients. RIT causes a transient depletion of B cells for approximately six to nine months. Severe and prolonged cytopenia, including both neutropenia and thrombocytopenia is common [2, 3].

Hematologic events during ofatumumab (CD20-directed MoAb), brentuximab vedotin (CD30-directed MoAb), and alemtuzumab (CD52-directed MoAb) treatment included anemia, neutropenia, and thrombocytopenia. Neutropenia (≥ grade 3: 42%; grade 4: 18%; may be prolonged >2 weeks) and anemia (16%; grades 3/4: 5%) have been reported in treatment with ofatumumab. No patients discontinued the drug due to AEs [45]. Grade 3/4 bone marrow suppression may occur in treatment with brentuximab vedotin, as shown by neutropenia (20%), thrombocytopenia (8%), and anemia (6%) [52–54]. Cytopenia in treatment with alemtuzumab includes the following: lymphopenia (grades 3/4: 97%), neutropenia (77%; grade 3/4: 42% to 64%), anemia (76%; grade 3/4: 12% to 38%), and thrombocytopenia (71%; grade 3/4: 13% to 52%). Serious and fatal cytopenia (including pancytopenia, bone marrow hypoplasia, autoimmune hemolytic anemia, and autoimmune idiopathic thrombocytopenia) has occurred. Single doses >30 mg or cumulative weekly doses >90 mg are associated with an increased incidence of pancytopenia [15, 41, 42, 90].

Treatment should be discontinued for serious hematologic or other serious toxicity (except lymphopenia) until the event resolves [45, 53, 90].

## 10. Other AEs

Progressive multifocal leukoencephalopathy (PML) due to JC virus infection has been reported with rituximab use, which may be fatal. Cases were reported in patients receiving rituximab. With combination chemotherapy, PML onset maybe delayed, although most cases were diagnosed within 12 months of the last rituximab dose. Clinical findings included confusion/disorientation, motor weakness/hemiparesis, altered vision/speech, and poor motor coordination with symptoms progressing over weeks to months. Cases of reversible posterior leukoencephalopathy syndrome (RPLS) have been reported with VEGF antibodies, which may be fatal. Symptoms of RPLS include headache, seizure, confusion, lethargy, blindness and/or other vision change, or neurologic disturbances. Some of the other less common AEs associated with therapeutic monoclonal antibodies used for cancer therapy include the following: fatigue, vomiting, abdominal pain, anorexia, dysphonia, and peripheral neuropathy [17, 33, 68, 74, 91]. Cetuximab and pannitumumab can induce magnesium wasting resulting in clinically significant hypomagnesemia/hypokalemia and hypokalemia [7, 11, 18].

## 11. Other MoAbs in Ongoing Clinical Trials

Two anti-PD-1 monoclonal antibodies, pembrolizumab and pidilizumab, have demonstrated activity in initial clinical trials in patients with advanced melanoma. Treatment AEs were manageable. The most common toxicities were fatigue, pruritus, rash, diarrhea, and arthralgia (36, 24, 20, 16, and 16%, resp.). Overall 12% of patients experienced grade 3 or 4 AEs [92, 93]. Anti-PD-1 monoclonal antibodies are currently being evaluated in randomized clinical trials. Clinical activity has been observed with several different anti-PD1-L1 monoclonal antibodies, including BMS-936559, MPDL3280A, BMS-936559, and MEDI4736, which has been evaluated in a dose escalation phase I trial with expansion cohorts in NSCLC, melanoma, and renal cell carcinoma [94, 95]. Further results from these studies are pending.

## 12. Summary

The panel of MoAbs that are approved by international regulatory agencies for the treatment of hematopoietic and solid malignancies has continued to expand. In this paper, we reviewed currently encompassing a stunning amount of 20 distinct molecules for 10 targets. We provide a brief scientific background on the use of MoAbs in cancer therapy, review all types of monoclonal antibodies-related adverse events (e.g., allergy, immune-related adverse events, cardiovascular adverse events, and pulmonary adverse events), and discuss the mechanism and treatment of adverse events (see Table 4).

Humanized monoclonal antibodies (MoAbs) have unique toxicities that differ from those of traditional chemotherapy. With the rapid development of targeted therapy to cancer, adverse events of MoAbs attract increasing attention. Further research is needed to explore the molecular mechanisms that underlie MoAb-related reactions to accurately identify hypersensitivity reactions and to develop new procedures for predicting AEs during MoAb treatment.

## Figures and Tables

**Table 1:** Monoclonal antibodies (MoAbs) approved for cancer therapy.

MoAb	Trade name	Target	Type	Indication(s)
Cetuximab	Erbitux	EGFR	Chimeric IgG1*κ*	HNC and colorectal cancer
Panitumomab	Vectibix	EGFR	Human IgG2*κ*	Colorectal carcinoma
Nimotuzumab	Nimotuzumab	EGFR	Human IgGh-R3	HNC
Bevacizumab	Avastin	VEGFR	Humanized IgG1*κ*	Colorectal, renal, lung, and brain cancer
Ramucirumab	Cyramza	VEGFR	Humanized IgG1*κ*	Gastric or gastresophageal junction cancer
Trastuzumab	Herceptin	HER2	Humanized IgG1*κ*	Breast cancer, gastric or gastroesophageal junction cancer
Trastuzumabemtansine	Kadcyla	HER2	Humanized IgG1*κ*	Breast cancer
Pertuzumab	Perjeta	HER2	Humanized IgG1*κ*	Breast cancer
Alemtuzumab	Campath	CD52	Humanized IgG1*κ*	Chronic lymphocytic leukemia
Rituximab	Rituxan MabThera	CD20	Chimeric IgG1*κ*	Chronic lymphocytic leukemia and non-Hodgkin lymphoma
Ofatumumab	Arzerra	CD20	Human IgG1*κ*	Chronic lymphocytic leukemia
Obinutuzumab	Gazyva	CD20	Human IgG1	CLL
Ibritumomab	Zevalin	CD20	Murine IgG1*κ*	Non-Hodgkin lymphoma
Tositumomab	Bexxar	CD20	Murine IgG2a*λ*	Non-Hodgkin lymphoma
Brentuximab Vedotin	Adcetris	CD30	Chimeric IgG1*κ*	Hodgkin's and anaplastic large cell lymphoma
Ipilimumab	Yervoy	CTLA-4	Human IgG1*κ*	Melanoma
Catumaxomab	Removab	EpCAM	Rat IgG2b/mouse IgG2a bispecific	Malignant ascites in patients with ePCaM + cancer
Denosumab	Prolia Xgeva	RANKL	Human IgG2*κ*	Breast cancer, prostate cancer, and giant cell tumors of the bone
Nivolumab	Opdivo	PD-1	Human IgG4	Melanoma
Siltuximab	Sylvant	IL-6	Chimeric IgG1*κ*	Castleman disease, multicentric (in patients who are HIV negative and HHV-8 negative)

**Table 2:** Clinical criteria for diagnosing anaphylaxis.

Anaphylaxis is highly likely when anyone of the following three criteria is fulfilled:	
(1) acute onset of an illness (minutes to several hours) with involvement of the skin, mucosal tissue, or both (e.g., generalized hives, pruritus or flushing, swollen lips, tongue, and uvula *and at least one of the following: *	
(a) respiratory compromise (e.g., dyspnea, wheeze-bronchospasm, stridor, reduced PEF, and hypoxemia),	
(b) reduced BP or associated symptoms of end-organ dysfunction (e.g., hypotonia [collapse], syncope, and incontinence);	
(2) two or more of the following that occur rapidly after exposure to a likely allergen for that patient (minutes to several hours):	
(a) involvement of the skin-mucosal tissue (e.g., generalized hives, itch-flush, and swollen lips, tongue, and uvula),	
(b) respiratory compromise (e.g., dyspnea, wheeze-bronchospasm, stridor, reduced PEF, and hypoxemia),	
(c) reduced BP or associated symptoms (e.g., hypotonia [collapse], syncope, and incontinence),	
(d) persistent gastrointestinal symptoms (e.g., crampy abdominal pain, vomiting);	
(3) reduced BP after exposure to known allergen for that patient (minutes to several hours):	
(a) infants and children: low systolic BP (age specific) or >30% decrease in systolic BP^*^	
(b) adults: systolic BP of <90 mmHg or >30% decrease from that person's baseline	

Notes

PEF, peak expiratory flow; BP, blood pressure.

^*^Low systolic blood pressure for children is defined as <70 mmHg from 1 month to 1 year, less than (70 mmHg + [2 × age]) from 1 to 10 years and <90 mmHg from 11 to 17 years.

**Table 3:** Emergency management: recommendations.

Recommendation	Evidence Level	Grade
**First-line intervention: adrenaline**		
Adrenaline is potentially lifesaving and must therefore promptly be administered as the first-line treatment for the emergency management of anaphylaxis.	IV	C
Earlier administration of adrenaline should be considered on an individual basis when an allergic reaction is likely to develop into anaphylaxis.	V	D
Adrenaline should be administered by intramuscular injection into the midouter thigh.	I	B
In patients requiring repeat doses of adrenaline, these should be administered at least 5 min apart.	V	D
With inadequate response to two or more doses of intramuscular adrenaline, adrenaline may be administered as an infusion by appropriately experienced intensive care, emergency department, and critical care physicians, with appropriate cardiac monitoring.	IV	D

**Second-line interventions**		
Trigger of the anaphylaxis episode should be removed.	V	D
Help should be called promptly and simultaneously with patient's assessment.	V	D
Patients experiencing anaphylaxis should be positioned supine with elevated lower extremities if they have circulatory instability, sitting up if they have respiratory distress, and in recovery position if unconscious.	V	D
High-flow oxygen should be administered by face mask to all patients with anaphylaxis.	V	D
Intravenous fluids (crystalloids) should be administered (boluses of 20 mL/kg) in patients experiencing cardiovascular instability.	V	D
Inhaled short-acting beta-2 agonists should additionally be given to relieve symptoms of bronchoconstriction.	V	D

**Third-line interventions**		
Oral H1- (and H2-) antihistamines may relieve cutaneous symptoms of anaphylaxis.	I	B
Systemic glucocorticosteroids may be used as they may reduce the risk of late-phase respiratory symptoms. High-dose nebulized glucocorticoids may be beneficial for upper airway obstruction.	V	D

**Monitoring and discharge**		
Patients who presented with respiratory compromise should be closely monitored for at least 6–8 h, and patients who presented with circulatory instability require close monitoring for 12–24 h.	V	D
Before discharge, the risk of future reactions should be assessed and an adrenaline autoinjector should be prescribed to those at risk of recurrence.	V	D
Patients should be provided with a discharge advice sheet, including allergen avoidance measures (where possible) and instructions for the use of the adrenaline autoinjector.	V	D
Specialist and food allergy specialist dietitian (in food anaphylaxis) followup should be organized. Contact information for patient support groups should also be provided.	V	D

**Table 4:** Adverse events of 20 MoAbs.

MoAb	Adverse events		Reference
	Systemic	Cutaneous	
Cetuximab	IR; cardiopulmonary arrest; GI; pulmonary toxicity; hypomagnesemia; infection; anaphylaxis	Rash/desquamation; acneiform rash; nail changes; pruritus; paronychial inflammation	[7, 11, 16, 17]

Panitumumab	IR; pulmonary fibrosis; electrolyte depletion; peripheral edema; GI; fatigue	Erythema; acneiform rash; pruritus; nail toxicity; exfoliation; paronychia skin fissures; photosensitivity	[18, 19]

Nimotuzumab	Fever; hypotension; tremor; lymphopenia,	Rash and chills	[20, 21]

Bevacizumab	Hypertension; VTE; ATE; GIP; hemorrhage; wound healing complications; fistula/abscess formation; CHF; IR; proteinurea; necrotizing fasciitis	Exfoliative dermatitis; xeroderma; alopecia	[22–27]

Ramucirumab	Hypertension; IR; ATE; GIP; hemorrhage; wound healing complications; RPIS	Skin rash	[28–30]

Trastuzumab	LVD;CHF; IR; pulmonary toxicity; neutropenia; anaphylaxis/angioedema; anemia; GI	Acne vulgaris; nail disorders; pruritus	[31–35]

Trastuzumabemtansine	Hepatotoxicity; LVD; pulmonary events; thrombocytopenia; neurotoxicity; hypersensitivity; IR; GI	Rash; pruritus	[36, 37]

Pertuzumab	IR; cytopenias; GI; PN; hypersensitivity/anaphylaxis; LVD	Alopecia; rash; paronychia; pruritus palmar-plantar erythrodysesthesia;, xeroderma; pruritus	[38–40]

Alemtuzumab	Cytopenias; IR; infections; immunogenicity; hypotension; hypertension; dysrhythmia; pulmonary events	Urticaria; rash; erythema;	[15, 41, 42]

Rituximab	IR; TLS; PML; renal toxicity; infections; cardiac events; pulmonary events; bowel obstruction/perforation; cytopenias; RA; anaphylaxis; HBr; SS; PML	Paraneoplastic pemphigus; rash; pruritus; angioedema; SJS; TEN	[5, 12–14, 43, 44]

Ofatumumab	IR; cytopenias; intestinal obstruction; PML; HBR; pneumonia; infections; dyspnea; diarrhea; PML; TLS	Rash; urticaria; hyperhidrosis	[45, 46]

Obinutuzumab	IR; hypocalcemia, hyperkalemia, hyponatremia; cytopenias; hepatic toxicity; infection; immunogenicity; HBR; PML; TLS	None	[47, 48]

Ibritumomab	IR; infections; severe cytopenias; immunogenicity; secondary malignancies; extravasation/radiation necrosis	EM; SJS; TeN; exfoliativedermatitis; rash;	[8, 49]

Tositumomab	Anaphylaxis; severe cytopenias; IR; fetal harm; hypothyroidism; secondary malignancies; infection	Rash; pruritus; sweating; dermatitis	[44, 50, 51]

Brentuximab Vedotin	PN; IR; cytopenias; TLS; infectionimmunogenicity; PML; anaphylaxis	SJS; rash; pruritus; alopecia	[52–55]

Ipilimumab	IrAEs; diarrhea; fatigue;	Dermatitis; pruritus; rash SJS; TEN	[56, 57]

Catumaxomab	SIRS; abdominal disorders; CRS; pyrexia; cytopenias	Rash; erythema; pruritus	[58]

Denosumab	Hypocalcemia; hypophosphatemia; embryo-fetal toxicity; ONJ and osteomyelitis; fatigue; dyspnea	Dermatitis; eczema; rash; pruritus	[59, 60]

Nivolumab	Fatigue; diarrhea; lymphopenia	Rash; pruritus; vitiligo	[61]

Siltuximab	GIP; IR; IR/hypersensitivity reactions; elevated hemoglobin levels; infection; diarrhea	Pruritus; skin rash	[62]

CRS, cytokine release syndrome; GI, gastrointestinal symptoms, for example, nausea, diarrhea, vomiting, and constipation; HBR, hepatitis B reactivation; IrAEs, immune-mediated reactions due to T cell activation and proliferation (enterocolitis, hepatitis, dermatitis, neuropathies, and endocrinopathies); IR, infusion reactions; LVD, left ventricular dysfunction; ONJ, osteonecrosis of the jaw; PML, progressive multifocal leukoencephalopathy; PN, peripheral neuropathy; SIRS, systemic inflammatory response syndrome; SJS, Stevens-Johnson syndrome; SS, serum sickness-like reactions; RPIS, reversible posterior leukoencephalopathy syndrome; TEN, toxic epidermal necrolysis;, TLS, tumor lysis syndrome.

## Abbreviations

ADCC: Antibody-dependent cellular cytotoxicity
AEs: Adverse events
ARDS: Acute respiratory distress syndrome
ATE: Arterial thromboembolic event
BALF: Bronchoalveolar lavage fluid
BOOP: Bronchiolitis obliterans organizing pneumonia
BSC: Best supportive care
CHF: Congestive heart failure
CTLA-4: Cytotoxic T lymphocyte-associated protein 4
CLL: Chronic lymphocytic leukemia
EGFR: Epidermal growth factor receptor
EpCAM: Epithelial cell adhesion molecule
FDA: Food and Drug Administration
HNC: Head and neck carcinoma
ILD: Interstitial lung disease
irAEs: Immune-related adverse events
LVEF: Left ventricular ejection fraction
MoAb: Monoclonal antibody
NHL: Non-Hodgkin's lymphoma
NSCLC: Non-small cell lung carcinoma
PD-1: Programmed death 1 protein
RPLS: Reversible posterior leukoencephalopathy syndrome
SIR: Standard infusion reaction
T-DM1: Trastuzumabemtansine
VEGF: Vascular endothelial growth factor
VTE: Venous thromboembolic event.
